# Editorial: Women in cellular neuropathology

**DOI:** 10.3389/fncel.2024.1423482

**Published:** 2024-05-09

**Authors:** Veronica Ghiglieri, Maria Teresa Viscomi

**Affiliations:** ^1^Department of Human Sciences and Quality of Life Promotion, Università Telematica San Raffaele, Rome, Italy; ^2^Fondazione Policlinico Universitario Agostino Gemelli IRCCS, Rome, Italy; ^3^Department of Life Sciences and Public Health, Università Cattolica del Sacro Cuore, Rome, Italy

**Keywords:** neurodegeneration, inflammation, glial cells, transdifferentiation, antimicrobial peptides, non-coding RNA

Neurological and psychiatric pathologies are debilitating diseases affecting millions worldwide, impacting the quality of life of both patients and their families, and requiring burdens on healthcare systems. These conditions, including Multiple Sclerosis and Parkinson's disease and ranging from schizophrenia to depression, present enormous challenges to the scientific community as they demand comprehensive research efforts to unravel their complex pathogenetic mechanisms.

In this editorial, we delve into the interconnectedness of these disorders through the lens of cellular and molecular biology, highlighting key research avenues and promising approaches.

At the forefront of neurological research lies the quest to decipher the cellular and molecular alterations underlying a disorder. Axonal damage, neuronal alterations, genetic variations, and abnormal glial activation stand as common hallmarks of neurodegenerative conditions. Recent studies, exemplified by the insights presented here, underscore the significance of decyphering these abnormalities throughout the disease progression for targeted therapeutic interventions.

The six articles collected in this Research Topic reflect the broad scope of current research on neurological and psychiatric diseases and provide a compelling overview of the cellular and molecular changes critical in neuronal degeneration. The methodologies employed and discussed in this Research Topic contribute unique insights, from animal models elucidating disease pathways to cellular transformation techniques enabling disease modeling (Joost et al.; Ng et al.; Laricchiuta et al.). Moreover, advanced imaging techniques enhance our understanding by providing detailed characterization of pathological alterations (Chapleau et al.) and bioactive molecules, including antimicrobial peptides and non-coding RNAs, emerge as pivotal players in the pathophysiology of neurological diseases potentially serving as therapeutic targets (Stuart et al.; Meccariello et al.).

Joost et al.s' research article elegantly investigated the myelin ultrastructural changes occurring at the axonal-myelin interface in a mice model of demyelination induced by metabolic oligodendrocyte injury. Intoxication with the copper-chelator cuprizone induces apoptotic cell death of oligodendrocytes within a few days, followed by the activation of the innate immune cells in the brain, i.e., astrocytes and microglia, leading to demyelination of distinct brain areas. In this model, the Authors showed the presence of myelin vacuolization due to the partial detachments of myelin from the axons. Such histopathological change that was evident, especially after 3 weeks of cuprizone intoxication, was concomitant to nodal and paranodal deterioration. This report demonstrates how the cuprizone model nicely recapitulates some striking aspects of progressive multiple sclerosis, mainly those occurring in early demyelination, showing its strength and reliability for studying early pathological changes during lesion formation.

The importance of histological examination for shedding light on the disease pathogenesis is underlined by Chapleau et al. in a case report of cavitating leukoencephalopathy caused by COA8 cytochrome C oxidase deficiency. In their study, the Authors report the clinical, neuroimaging, and neuropathological results of a young patient with cavitating mitochondrial leukoencephalopathies caused by pathogenic 124 variants encoding subunits of complex I, such as NDUFV1and NDUFA1. Such a rare disease is a neurodegenerative disorder characterized by acute neurologic deficits and progressive or intermittent functional deterioration. More interestingly, postmortem histological examinations show some relevant pathology features, such as myelin damage, macrophage infiltration at the sites of demyelination, and extensive astrogliosis. Characterizing brain histopathological hallmarks is crucial to clarify disease-specific patterns, elucidate causative mechanisms relevant to rare diseases, and provide clues into molecular mechanisms contributing to the clinical features observed. Morphological changes observed in pathological conditions are related to distinct underlying molecular profiles that drive cellular structure and function.

In their review, Meccariello et al. exhaustively discuss how non-coding RNA (ncRNA), including long-non-coding RNA (lncRNA), microRNA (miRNA), and circular-RNA (circRNA), contribute to the molecular events underlying progressive neuronal degeneration, mainly focusing on Parkinson's disease and synucleinopathy. ncRNAs represent a hot topic of research as they may be valuable tools for diagnosis, prognosis, and therapeutic biomarkers for different pathologies. In brain diseases characterized by synucleinopathy, RNA, and miRNA have been proposed as possible molecular mechanisms causing synaptic and neuronal dysfunction. Indeed, since they are small molecules permeable to the brain-blood barrier, they can be transported to synapses, where they control local protein synthesis and target gene expression crucial for synaptic plasticity. ncRNA are crucial mediators of inflammatory responses associated with neurodegeneration, as they regulate, beyond their classical roles in gene silencing, the expression of proinflammatory cytokines and metabolic activities in macrophages during inflammation. This review on the ncRNA involvement in PD and synucleinopathies elucidates complex aspects of the disease pathogenesis and dwells on their role as molecular signatures that can be validated for prediction, diagnosis, prognosis, and therapy in PD pathogenesis.

Stuart et al., in their review, discuss recent literature on a new field of study associated with neurodegeneration. They highlight the crosstalk between Antimicrobial Peptides (AMPs) and the nervous system at both molecular and functional levels. AMPs are short cationic peptides able to fight off pathogens. However, recent evidence highlights the multifunctional roles of AMPs, including their involvement in regulating neurons and the bidirectional interactions between the nervous system and non-neuronal tissues.

In the paper, the Authors underline the role of AMPs in neuroinflammation, a common histopathological feature of aging, psychiatric and neurodegenerative diseases. In several pathologies, the activation of Toll-like receptors (TLRs), a family of transmembrane proteins, upon stress or damage, triggers an inflammatory response—aimed to eliminate the bacteria and repair damaged tissues—that includes a series of inflammatory signaling molecules as well AMPs.

In the central nervous system, AMPs act as double-edged sword molecules as they can be essential mediators of inflammation—by stimulating glial cells to release proinflammatory cytokines—or act as anti-inflammatory molecules—by preventing the activation of TLR2 and TLR4. Since little is known about this topic, the Authors claim the need for further efforts to clarify whether AMPs are the drivers of brain diseases or whether their altered function is a consequence of the progression of these diseases. Answering these critical questions will help identify important biomarkers for brain pathologies and contribute to developing novel therapeutic targets.

Neuroinflammation is a physiological and necessary process tightly regulated in response to brain injury or stress. Triggered by various internal and external stimuli, inflammation is controlled by the immune system, which, through a series of signaling pathways, helps restore brain homeostasis. However, dysregulation of inflammatory responses can trigger brain disease. Abnormal immune function contributes to the development and progression of neurodegenerative diseases as well as psychiatric disorders. However, the exact molecular mechanisms underlying the link between these pathophysiological factors and psychiatric disorders have not been clarified yet.

In their review, Laricchiuta et al. provide an interesting overview of the aberrant responses of glial cells, the leading players in the induction and execution of inflammation in the central nervous system, in the context of psychiatric diseases such as schizophrenia. In schizophrenia, as in other psychiatric diseases, the overactivated glial cells contribute to dysregulating neuron-glia crosstalk by impairing neurotransmission and brain homeostasis, ultimately impacting cognitive functions. They summarized evidence from different experimental *in vivo* and *ex vivo* models and patients, showing the link between glia alterations—such as changes in the morphology, density, and function—and the development or the presence or severity of symptoms in the case of patients with schizophrenia. Although we are still far from identifying the precise glia-associated mechanisms in the pathophysiology of the disease, the relevance of inflammation suggests its potential as a suitable biomarker for diagnosing the disease and, ultimately, for its treatment.

For human disease characterization, molecular and cellular research uses experimental models—*in vitro, ex vivo*, and *in vivo*—to investigate disease processes and test potential therapeutics. In this field, in recent years, with the improvement of direct cell reprogramming technology, it has become possible to induce non-neuronal cells, such as fibroblasts, directly into neuronal cells.

In their review, Ng et al. discuss techniques used for rapid and efficient conversion of somatic cells—such as fibroblast and blood cells—into neural cell types and their importance in the field of disease modeling. Their mini-review presents strengths, limitations, and future perspectives of this rapidly revolutionary field that could boost the study of human live cell models for brain pathologies and potentiate the development of tailored therapies for different pathologies.

In conclusion, this Research Topic provides evidence that understanding the molecular and cellular basis of neurological and psychiatric disorders is crucial for tailored diagnosis and treatment.

As we embark on this collective journey of discovery, the commitment should be to combine new methodological approaches for developing multi-targeted drug combinations and translate our findings into tangible benefits with disease-modifying therapies.

By integrating insights from different research avenues, we can forge new paths toward improved patient outcomes and ultimately alleviate the burden of these debilitating conditions.

## Author contributions

VG: Conceptualization, Data curation, Resources, Writing – original draft, Writing – review & editing. MTV: Supervision, Validation, Visualization, Writing – original draft, Writing – review & editing.

